# Different Habitat Types Affect Bird Richness and Evenness

**DOI:** 10.1038/s41598-020-58202-4

**Published:** 2020-01-27

**Authors:** Hung-Ming Tu, Meng-Wen Fan, Jerome Chie-Jen Ko

**Affiliations:** 1 Department of Horticulture, National Chung Hsing University, Taichung, 40227 Taiwan; 2 Endemic Species Research Institute, Nantou, 55244 Taiwan

**Keywords:** Grassland ecology, Agroecology, Biodiversity, Forest ecology, Urban ecology

## Abstract

More studies are needed on the mechanism and effective prediction of bird diversity in various habitats. The primary purpose of this study was to explore the difference in the species richness and evenness of various habitats. The secondary purpose was to explore which habitat types and compositions predict a high bird diversity. The 2010–2016 Taiwan Breeding Bird Survey was used to analyze the relationship between landscape habitat and bird ecology. Landscape habitat type was divided into seven categories and 26 sub-types: forestland, farmland, grassland, freshwater wetland, aquaculture pond and saltpan, coastland, and building area. Four ecological indexes were used: the number of bird individuals, the number of species, the Margalef Richness Index, and the Pielou Evenness Index. The result indicated that forestland decreased bird numbers, except in a windbreak forest. Natural and farmland-related habitats increased bird species richness. Similarly, the natural habitat increased species evenness. Urban greenspace could not replace the effect of forestland on species richness and evenness. Conifer forest, bamboo forest, windbreak forest, mixed tree, tall grassland, and orchard were important habitats for promoting higher species richness and evenness.

## Introduction

The relationship between various environments and bird diversity has been a critical issue. A large number of studies have explored the variety of bird diversity in urban and rural areas^1–8^, farmland^9–12^, and forestland^13–16^. Some studies indicated that a higher ecological diversity not only benefits species survival but is also an important indicator of human well-being^17,18^. The promotion of bird diversity is a useful method for generating human psychological benefits^19^. Therefore, the mechanism and effective prediction of bird diversity in various habitats should be understood.

The mechanisms relating to various habitats and bird diversity with human activity are still not clear. Species richness and species evenness are two common concepts to measure species diversity^20^. The number of breeding bird species increases from urban to suburban, rural, and natural areas^2,4,12,21^. A few studies indicated that urbanization did not reduce bird species richness (i.e., the number of bird species) due to an abundant food supply, but rather increased the number of birds in a few dominant bird species^22^. One of the main characteristics of urban areas is the numerical dominance of a few abundant bird species^21^, which means a lower species evenness. This observation corresponds to the primary purpose of the study: species evenness may demonstrate a dissimilarity in bird diversity between natural and urban environments. Few studies have separately explored bird species richness and evenness to answer this question in various bird habitats.

Some habitat types and characteristics have been studied and have shown positive results for higher bird diversity. The presence of forest is a positive environmental characteristic for bird diversity in various environments^3,5,7,8,12–14,23^. The percentage of coniferous tree cover is an important variable for predicting the number of birds^8^ and bird species richness^2^. Mixed tree species are critical for attracting bird species^24^. Mixed conifer-deciduous forests had a higher bird species richness^2,23^. Some studies indicated similar results for bird diversity in mixed conifer-deciduous forests: the study of Fontana *et al*. indicated that a mixed conifer-broadleaf forest had no influence on the bird Simpson’s diversity index, but had an influence on bird community composition^2^. Agroforests may be an important habitat for bird diversity because they attract fruit-eating species^14^. A few studies indicated that orchards had no or low effect on the bird species diversity^10,11^. Contrarily, other studies reported a similarity between the bird diversity of orchards and primary forests^9^. There is no consistent relationship between various habitats and bird diversity. This observation corresponds to the secondary purpose of this study: identifying which habitat types and composition predict a higher bird diversity. Bird habitats include various habitat types: from the human-related environment (e.g., building area, park, rural area, farmland, and pond) to the natural-related environment (e.g., forestland, grassland, river, stream, and coastland).

More studies are needed to predict bird diversity in different habitat types. The primary purpose of this study was to explore bird diversity in various habitats, including species richness and evenness. The second purpose was to examine which habitat types and compositions predict a high bird diversity.

## Results

A total of 31,442 records of single bird detection, 295 bird species, and 540,254 birds (in number) had been documented during 2010 to 2016, including breeding birds and non-breeding migratory birds. The entire list of 295 bird species can be found as Supplementary Table S1 online. Of these, 786 were excluded due to missing information on landscape type, wind, and weather. A total of 30,656 valid records were obtained for data analysis. The mean (S.D.) number of individual birds and species at each sampling point was 17.23 (16.48) and 5.93 (2.86), respectively (Table 1). Forestland (58.4%), farmland (33.8%), grassland (19.3%), building area (18.5%), and freshwater wetland (14.2%) were main landscape habitat types (Table 2). Broadleaf forest (36.2%), orchard (16.7%), aquatic farmland (12.0%), mixed bamboo–broadleaf forest (10.9%), tall grassland (10.9%), dry farmland (9.7%), and mixed conifer–broadleaf forest (9.1%) were the main landscape sub-habitats.

**Table 1:** Descriptive statistics for bird diversity (n = 30,656).

Index	Min.	Max.	Mean	S.D.
Number of bird individuals (N)	1.00	466.00	17.23	16.48
Number of species (S)	1.00	23.00	5.93	2.86
Margalef Richness Index (d)	0.00	5.19	1.83	0.83
Pielou Evenness Index (J’)	0.00	1.00	0.84	0.21

**Table 2:** Descriptive statistics for landscape habitat types and sub-types. Each survey sampling point recorded one or two main landscape habitat types and sub-types. The number of landscape habitat types and sub-types were higher than 30,656.

The type of landscape habitat	n	%
Forestland	17,899	58.4
Broadleaf forest	11,099	36.2
Conifer forest	884	2.9
Mixed conifer–broadleaf forest	2,790	9.1
Bamboo forest	1,121	3.7
Mixed bamboo–broadleaf forest	3,349	10.9
Windbreak forest	316	1.0
Grassland	5,905	19.3
Tall grassland (height >0.5 m)	3,356	10.9
Low grassland (height <0.5 m)	1,557	5.1
High marsh (height >0.5 m)	563	1.8
Low marsh (height <0.5 m)	214	0.7
Bamboo grassland	307	1.0
Freshwater wetland	4,344	14.2
Water storage area	387	1.3
Lake (natural)	378	1.2
River (water surface width >3 m)	2,638	8.6
Stream (water surface width <3 m)	949	3.1
Coastland	237	0.8
Intertidal zone	140	0.5
Shoreline	102	0.3
Farmland	10,353	33.8
Aquatic farmland	3,674	12.0
Dry farmland	2,981	9.7
Orchard	5,111	16.7
Aquaculture pond and saltpan	784	2.6
Flooded field	705	2.3
Dried field	74	0.2
Abandoned field	80	0.3
Building area	5,679	18.5
Urban park and greenspace	2,027	6.6
Urban building area	1,323	4.3
Rural building area	2,643	8.6

## The Prediction of Bird Diversity by Habitat Type

The unstandardized and standardized coefficients of habitat types (dummy variable) on bird diversity through regression analysis is shown in Table 3. Grassland, freshwater wetland, farmland, aquaculture pond and saltpan, and building area significantly increased the number of birds by 1.64 (*B* = 0.04, *p* < 0.001), 0.95 (*B* = 0.02, *p* < 0.001), 5.35 (*B* = 0.15, *p* < 0.001), 17.32 (*B* = 0.17, *p* < 0.001), and 3.19 (*B* = 0.08, *p* < 0.001) birds, respectively. However, forestland significantly decreased the number of birds by 2.85 (*B* = −0.09, *p* < 0.001). Forestland, grassland, freshwater wetland, farmland, aquaculture pond and saltpan, and building area significantly increased the number of bird species by 0.29 (*B* = 0.05, *p* < 0.001), 0.64 (*B* = 0.09, *p* < 0.001), 0.51 (*B* = 0.06, *p* < 0.001), 1.06 (*B* = 0.04, *p* < 0.001), 1.11(*B* = 0.11, *p* < 0.001), and 0.16 (*B* = 0.02, *p* < 0.01), respectively. Only coastland significantly decreased the number of bird species by 1.06 (*B* = −0.03, *p* < 0.001).

**Table 3:** Effect of the landscape habitat type on the diversity index. Control variables already included in model 1. N; number of birds; S: number of species; d: Margalef Richness Index; J’: Pielou Evenness Index. * p < 0.05; **p < 0.01; ***p < 0.001.

	Diversity index							
	N		S		d		J'	
	Model 2		Model 2		Model 2		Model 2	
	B(SE)	Beta	B(SE)	Beta	B(SE)	Beta	B(SE)	Beta
Constant	14.94(0.31)		5.25(0.06)		1.65(0.02)		0.83(0.00)	
**Control variable**								
Wind	0.06(0.16)	0.00	−0.50(0.03)	−0.10^***^	−0.16(0.01)	−0.11^***^	−0.02(0.00)	−0.06^***^
Cloudy	0.87(0.22)	0.02^***^	0.21(0.04)	0.03^***^	0.04(0.01)	0.02^**^	0.00(0.00)	0.00
Overcast	1.46(0.23)	0.04^***^	0.15(0.04)	0.02^***^	0.02(0.01)	0.01	0.00(0.00)	−0.01
Dense fog	−0.01(0.69)	0.00	−0.06(0.12)	0.00	0.00(0.04)	0.00	−0.01(0.01)	−0.01
**Independent variable**								
Forestland	−2.85(0.26)	−0.09^***^	0.29(0.05)	0.05^***^	0.20(0.01)	0.12^***^	0.03(0.00)	0.07^***^
Grassland	1.64(0.25)	0.04^***^	0.64(0.04)	0.09^***^	0.17(0.01)	0.08^***^	0.02(0.00)	0.03^***^
Freshwater wetland	0.95(0.28)	0.02^***^	0.51(0.05)	0.06^***^	0.14(0.01)	0.06^***^	0.00(0.00)	0.00
Coastland	−1.71(1.05)	−0.01	−1.08(0.19)	−0.03^***^	−0.35(0.05)	−0.04^***^	−0.05(0.01)	−0.02^***^
Farmland	5.35(0.24)	0.15^***^	1.06(0.04)	0.18^***^	0.20(0.01)	0.11^***^	0.00(0.00)	0.00
Aquaculture pond and saltpan	17.32(0.59)	0.17^***^	1.11(0.11)	0.06^***^	0.05(0.03)	0.01	−0.03(0.01)	−0.03^***^
Building area	3.19(0.28)	0.08^***^	0.16(0.05)	0.02^**^	−0.06(0.01)	−0.03^***^	−0.01(0.00)	−0.03^***^
R^2^		0.082		0.039		0.035		0.014
ΔR^2^		0.079		0.030		0.023		0.010
adj R^2^		0.082		0.038		0.035		0.014

Forestland, grassland, freshwater wetland, and farmland significantly increased the Richness Index by 0.20 (*B* = 0.12, *p* < 0.001), 0.17 (*B* = 0.08, *p* < 0.001), 0.14 (*B* = 0.06, *p* < 0.001), and 0.20 (*B* = 0.11, *p* < 0.001), respectively. Coastland and building area significantly decreased the Richness Index by 0.35 (*B* = −0.04, *p* < 0.001) and 0.06 (*B* = −0.03, *p* < 0.001), respectively. Forestland and grassland significantly increased the Evenness Index by 0.03 (*B* = 0.07, *p* < 0.001), and 0.02 (*B* = 0.03, *p* < 0.001), respectively. Coastland, aquaculture pond and saltpan, and building area significantly decreased the Evenness Index by 0.05 (*B* = −0.02, *p* < 0.001), 0.03 (*B* = −0.03, *p* < 0.001), and 0.01 (*B* = −0.03, *p* < 0.001), respectively.

The results indicate that forestland and grassland significantly increased the number of bird species, richness, and evenness, although the presence of forestland significantly decreased bird numbers. Farmland and freshwater wetlands significantly increased bird numbers, the number of bird species, and richness, but did not affect evenness. Aquaculture ponds and building area significantly increased bird numbers and the number of bird species, but significantly decreased species evenness, which means that the number of birds per bird species was not equal. Coastland decreased bird species, richness, and evenness.

## The Prediction of Bird Diversity by Habitat Sub-Type

The regression analysis of habitat sub-type influence on bird diversity is shown in Table 4. The results indicated that forestland sub-types significantly increased the number of bird species, richness, and evenness, except for broadleaf forest. Broadleaf forest only increased species richness (*B* = 0.03, *p* < 0.001). The bird number was significantly decreased in the broadleaf forest (*B* = 0.07, *p* < 0.001) and mixed conifer–broadleaf forest (*B* = 0.03, *p* < 0.001), and significantly increased in the windbreak forest (*B* = 0.09, *p* < 0.001). The windbreak forest significantly increased the values of all four indexes, especially increasing the bird numbers and numbers of species by 14.62 and 2.10, respectively. The forestland sub-type did not have high bird numbers, except in the windbreak forest, but had high species evenness compared to other sub-types. Although the values of richness and evenness in the forestland sub-type were similar to the mixed forest sub-type, the mixed forest had slightly higher values.

**Table 4:** Effect of the landscape habitat sub-type on the diversity index. Control variables already included in model 1. N; number of birds; S: number of species; d: Margalef Richness Index; J’: Pielou Evenness Index. *p < 0.05; **p < 0.01; ***p < 0.001.

	Diversity index							
	N		S		d		J'	
	Model 2		Model 2		Model 2		Model 2	
	B(SE)	Beta	B(SE)	Beta	B(SE)	Beta	B(SE)	Beta
Constant	14.04(0.33)		5.24(0.06)		1.69(0.02)		0.84(0.00)	
**Control variable**								
Wind	0.01(0.16)	0.00	−0.49(0.03)	−0.10^***^	−0.16(0.01)	−0.11^***^	−0.02(0.00)	−0.06^***^
Cloudy	0.91(0.22)	0.03^***^	0.20(0.04)	0.03^***^	0.04(0.01)	0.02^**^	0.00(0.00)	0.00
Overcast	1.36(0.23)	0.04^***^	0.14(0.04)	0.02^***^	0.02(0.01)	0.01	0.00(0.00)	−0.01
Dense fog	0.07(0.69)	0.00	−0.07(0.12)	−0.01	−0.01(0.04)	0.00	−0.01(0.01)	−0.01
**Independent variable**								
**Forestland**								
Broadleaf forest	−2.47(0.27)	−0.07^***^	−0.06(0.05)	−0.01	0.05(0.01)	0.03^***^	0.01(0.00)	0.03
Conifer forest	0.01(0.62)	0.00	0.90(0.11)	0.05^***^	0.29(0.03)	0.06^***^	0.03(0.01)	0.03^***^
Mixed conifer–broadleaf forest	−1.97(0.39)	−0.03^***^	0.47(0.07)	0.05^***^	0.24(0.02)	0.08^***^	0.04(0.01)	0.05^***^
Bamboo forest	0.43(0.49)	0.00	0.75(0.09)	0.05^***^	0.24(0.03)	0.06^***^	0.03(0.01)	0.03^***^
Mixed bamboo–broadleaf forest	−0.31(0.34)	−0.01	0.61(0.06)	0.07^***^	0.22(0.02)	0.08^***^	0.03(0.00)	0.04^***^
Windbreak forest	14.62(0.93)	0.09^***^	2.10(0.16)	0.07^***^	0.49(0.05)	0.06^***^	0.04(0.01)	0.02^**^
**Grassland**								
Tall grassland (height >0.5 m)	2.52(0.31)	0.05^***^	1.09(0.05)	0.12^***^	0.28(0.02)	0.11^***^	0.02(0.00)	0.03^***^
Low grassland (height <0.5 m)	2.00(0.43)	0.03^***^	0.23(0.08)	0.02^**^	0.01(0.02)	0.00	0.00(0.01)	0.00
High marsh (height >0.5 m)	3.10(0.69)	0.03^***^	0.53(0.12)	0.02^***^	0.07(0.04)	0.01	0.02(0.01)	0.02^**^
Low marsh (height <0.5 m)	3.41(1.09)	0.02^**^	0.24(0.19)	0.01	−0.04(0.06)	0.00	0.00(0.01)	0.00
Bamboo grassland	−5.53(1.01)	−0.03^***^	−0.59(0.18)	−0.02^**^	0.07(0.05)	0.01	0.05(0.01)	0.03^***^
**Freshwater wetland**								
Water storage area	2.61(0.81)	0.02^**^	0.76(0.14)	0.03^***^	0.16(0.04)	0.02^***^	−0.01(0.01)	0.00
Lake (natural)	3.26(0.82)	0.02^***^	0.24(0.15)	0.01	0.00(0.04)	−0.00	−0.03(0.01)	−0.02^**^
River (water surface width >3 m)	1.77(0.35)	0.03^***^	0.76(0.06)	0.07^***^	0.21(0.02)	0.07^***^	0.01(0.00)	0.01
Stream (water surface width <3 m)	−0.47(0.53)	0.00	0.34(0.09)	0.02^***^	0.10(0.03)	0.02^***^	0.01(0.01)	0.01
**Coastland**								
Intertidal zone	0.53(1.35)	0.00	−0.76(0.24)	−0.02^**^	−0.28(0.07)	−0.02^***^	−0.01(0.02)	0.00
Shoreline	−7.09(1.58)	−0.02^***^	−1.67(0.28)	−0.03^***^	−0.49(0.08)	−0.03^***^	−0.10(0.02)	−0.03^***^
**Farmland**								
Aquatic farmland	6.73(0.33)	0.13^***^	0.45(0.06)	0.05^***^	−0.03(0.02)	−0.01	−0.03(0.00)	−0.04^***^
Dry farmland	5.03(0.34)	0.09^***^	0.96(0.06)	0.10^***^	0.17(0.02)	0.06^***^	0.00(0.00)	0.00
Orchard	3.13(0.28)	0.07^***^	1.13(0.05)	0.15^***^	0.27(0.01)	0.12^***^	0.02(0.00)	0.04^***^
**Aquaculture pond and saltpan**								
Flooded field	13.64(0.66)	0.12^***^	0.77(0.12)	0.04^***^	−0.03(0.03)	−0.01	−0.04(0.01)	−0.03^***^
Dried field	19.75(1.87)	0.06^***^	0.15(0.33)	0.00	−0.19(0.10)	−0.01^*^	−0.06(0.02)	−0.01^*^
Abandoned field	−0.34(1.8)	0.00	0.85(0.32)	0.02^**^	0.26(0.09)	0.02^**^	0.04(0.02)	0.01
**Building area**								
Urban park and greenspace	3.89(0.41)	0.06^***^	0.08(0.07)	0.01	−0.10(0.02)	−0.03^***^	−0.02(0.01)	−0.02^***^
Urban building area	0.21(0.48)	0.00	−0.26(0.09)	−0.02^**^	−0.14(0.02)	−0.03^***^	−0.01(0.01)	−0.01
Rural building area	5.24(0.35)	0.09^***^	0.65(0.06)	0.06^***^	0.05(0.02)	0.02^*^	−0.01(0.00)	−0.01
R^2^		0.101		0.065		0.060		0.020
ΔR^2^		0.098		0.055		0.047		0.016
adj R^2^		0.100		0.064		0.059		0.019

In grassland sub-types, tall grassland significantly increased the value of all indexes. High marsh significantly increased bird species evenness (*B* = 0.02, *p* < 0.01). Low grassland and low marsh did not influence the bird species richness and evenness. Also, grassland sub-types led to a significant increase in bird numbers and number of species, except for bamboo grassland. Although bamboo grassland significantly decreased bird numbers with a factor 5.53 (*B* = −0.03, *p* < 0.001) and the number of species with a factor 0.59 (*B* = −0.02, *p* < 0.01), there was a significant increase in bird species evenness (*B* = 0.03, *p* < 0.001). The results indicated that the height of grassland had a positive effect on species evenness, as greater heights is the common feature of tall grassland, high marsh, and bamboo grassland.

A stream significantly increased the number of species (*B* = 0.02, *p* < 0.001) and the species richness (*B* = 0.02, *p* < 0.001) in freshwater wetland sub-types. River and water storage areas significantly increased bird numbers, the number of species, and species richness, but did not influence species evenness. Although a lake significantly increased bird numbers (*B* = 0.02, *p* < 0.001), a significant decrease in species evenness (*B* = −0.02, *p* < 0.01) was found. An intertidal zone significantly decreased the number of species (*B* = −0.02, *p* < 0.01) and species richness (*B* = −0.02, *p* < 0.001) in coastland sub-types. The presence of a shoreline significantly decreased the value of all indexes. The coastland sub-type led to lower bird species richness and evenness.

Among the farmland sub-types, an orchard significantly increased the value of all indexes. Dry farmland significantly increased bird numbers (*B* = 0.09, *p* < 0.001), the number of species (*B* = 0.10, *p* < 0.001), and species richness (*B* = 0.06, *p* < 0.001), but did not influence species evenness. Although an aquatic farmland significantly increased bird numbers (*B* = 0.13, *p* < 0.001) and the number of species (*B* = 0.05, *p* < 0.001), there was a significant decrease in species richness. In the aquaculture pond and saltpan sub-type, only abandoned fields significantly increased bird species richness (*B* = 0.02, *p* < 0.01), while dried field significantly decreased bird species richness (*B* = −0.01, *p* < 0.05). Flooded fields and dried fields significantly decreased bird species evenness (*B* = −0.03, *p* < 0.001 and *B* = −0.01, *p* < 0.05, respectively).

Among the three sub-types of building areas, rural building areas significantly increased bird numbers (*B* = 0.09, *p* < 0.001), the number of species (*B* = 0.06, *p* < 0.001), and species richness (*B* = 0.02, *p* < 0.05), but did not influence species evenness. Only rural building areas significantly increased bird species richness in building area sub-types. The urban building area significantly decreased the number of species (*B* = −0.02, *p* < 0.01) and species richness (*B* = −0.03, *p* < 0.001), but did not influence the bird numbers and species evenness. Urban parks and greenspaces significantly increased bird numbers (*B* = 0.06, *p* < 0.001), significantly decreased bird species richness (*B* = −0.03, *p* < 0.001) and species evenness (*B* = −0.02, *p* < 0.001), and did not influence the number of species. Both the urban building area and urban parks and greenspaces demonstrated a similar significant decrease in species richness. The higher bird number and lower species evenness in urban parks and greenspaces were dissimilar to the urban building area.

## Discussion

The results showed that bird species richness and evenness were different between natural and human-related habitats. Three detailed characteristics can be identified. First, a lower bird number was a main forest characteristic because in most other natural, farmland-related, and human-related habitat types, except coastland, there was an increase in the number of birds. Urban areas often showed a higher bird density in past studies^22^. This study found that high bird numbers were not only present in building areas and farmlands but also in grasslands, freshwater wetlands, aquaculture ponds, and saltpans. Forestland and grassland demonstrated an increase in species richness and evenness. The dissimilarity in the number of birds between forestland and grassland could be explained by food availability. Grasslands may have higher food availability, which is the ultimate determinant of variation in local bird density^22,25^. Natural habitats had a higher positive effect on bird species evenness compared to farmland-related and urban habitats.

Forestland, grassland, freshwater wetland, and farmland had a positive effect on species richness. Building areas decreased species richness. Therefore, the second main characteristic was an increased bird species richness in natural and farmland-related habitats. Forestland sub-types had a similar positive effect on bird species richness. Parts of natural and farmland-related habitats, including tall grassland, river, stream, dry farmland, orchard, and abandoned fields had a positive effect on bird species richness. These environments reflected a higher food availability to promote bird species richness^25^; the higher food availability in the orchard habitat had the highest effect on bird species richness and the seasonal change in primary productivity altered bird species richness^26^. In the study of Waltert *et al*., farmland and forestland had a similar bird species richness, and frugivorous and omnivorous bird species richness did not differ between these habitats^27^. Urban building areas and urban parks and greenspaces had a negative effect on species richness. Vegetation is an important factor because mature trees and shrubs are valuable food resources for birds in urban areas^1,28^. In summary, natural and farmland-related habitats had a higher bird species richness than urban habitats.

The third main characteristic was an increased bird species evenness in natural habitats. Past studies have shown that urban green areas have higher evenness values compared to other urban areas^4^. The results showed that only forestland and grassland had an increased effect on bird species evenness in natural habitats. Three habitat types had a negative effect on bird species evenness: coastland, aquaculture pond and saltpan, and building area. In the analysis of sub-types, only the orchard had an increased effect on bird species evenness in farmland-related habitats. A past study found that bird species evenness was lowest in cultivated land and highest in a national conservation area^29^. Therefore, most of the areas with increased bird species evenness were natural habitats.

The results showed that five forestland sub-types effectively predict bird species richness and evenness: conifer forest, mixed conifer–broadleaf forest, bamboo forest, mixed bamboo–broadleaf forest, and windbreak forest. Mixed trees, conifer forests, and bamboo forests were important characteristics to promote species richness and evenness. Interestingly, broadleaf forests did not affect bird species evenness, but mixed broadleaf forests had a positive effect. The broadleaf forest was close to various human activities (human disturbance), possibly complicating the relationship between habitat and bird ecology. For example, forest openings both reduce nest success and increase nest density for some bird species^30^. In human-related areas, bird species richness values are sensitive to site-specific habitat characteristics^4^. The effect of forest maturity and fragmentation was also a possible factor affecting species richness and evenness^31^. This study indicated that bamboo forests and windbreak forests had a positive effect on bird species richness and evenness. The anthropogenic forest edge may create attractive habitats and have higher bird densities due to the heterogeneity of edge vegetation^30,32^. According to the study of Wuczyński, shrubby margins were particularly useful predictors of bird diversity^12^. The study showed that windbreak forests had a higher bird number, species number, species richness, and species evenness than other forest types.

Tall grasslands, streams, and orchards increased bird species richness and evenness. A past study indicated that breeding bird species richness significantly increased with grassland size^33^. In the study of Herzog *et al*., the pasture grassland did not contribute to bird diversity^10^. Contrarily, the results of this study showed that grasslands increased bird species richness and evenness. Of the five grassland sub-types, tall grasslands, tall marshes, and bamboo grasslands had the most positive effect on bird species evenness. Only the tall grassland increased both bird species richness and evenness. Tall grassland is higher than 0.5 m. The height of grassland may be the critical characteristic for bird species evenness because tall marsh and bamboo grassland also have greater height and higher species evenness. Few studies have explored the effect of bamboo grassland on bird diversity. The results indicated that bamboo grassland had some unique characteristics: increased bird species evenness and decreased bird number and number of bird species richness.

A past study indicated that a river corridor in freshwater wetlands had a higher species richness in the valley area^6^. This study divided the river corridor into river (water surface width greater than 3 m) and stream (water surface width smaller than 3 m). The results showed that the river, stream, and water storage areas increased species richness. The river had the highest effect on species richness among the freshwater wetland sub-types. The water surface width of the river should be considered in river design and restoration. The lake habitat had no influence on species evenness. In a study by Yuan *et al*., sedge area, water area, reed area, patch density, and distance to residents were important characteristics of bird species abundance^34^. Landscape structure and human disturbance may affect the results of this study and should be considered and clarified in future studies. In the coastland sub-type, the intertidal zone and shoreline did not benefit bird species richness and evenness. Coastland is a unique habitat that differs from forests and urban habitats and likely attracts unique bird communities.

The orchard highly contributed to species richness and evenness in the farmland habitat. This study supported the result of Beukema *et al*., stating that the bird diversity of orchards was similar to that of primary forests^9^. Orchard can be considered a critical ecological habitat for rural farmland. Dry farmland increased species richness. However, aquatic farmland decreased species evenness, meaning that bird communities in aquatic farmland are dominated by certain species. In aquaculture ponds and saltpans, flooded and dried fields are also dominated by specific species. The abandoned fields increased species richness. The reduction of human disturbance could support the restoration of the bird ecology.

The rural building areas increased the bird number and species richness. However, the results of this study showed that urban areas, including urban parks and greenspaces, reduced species richness. Past studies showed that urbanisation increased the number of birds for a few dominant bird species and did not reduce bird species richness^22^. Some studies also showed that urban greenspaces had a higher species richness than urban areas^7^. Urban parks and greenspaces had three characteristics in this study: increasing bird numbers and decreasing species richness and evenness. The critical factors may be park size^7^, mature tree proportion^1^, and number of trees with cavities^7^, meaning that urban parks and greenspaces could not replace forestlands for bird diversity. However, these factors have been studied insufficiently.

The current study included some limitations. The first limitation is the data structure of the landscape habitat. The secondary data from Taiwan Breeding Bird Survey (BBS Taiwan) recorded two main landscape habitat types through the categorical variable. Therefore, the landscape habitat was recorded into the dummy variable in the data analysis process, which makes it challenging to explain the relationship of continuous dependent variables. The second limitation is investigator bias. BBS Taiwan surveyed bird data with the help of volunteer surveyors. Although field excursions and training workshops were held to ensure that surveyors fully understood the survey method, the individual differences between surveyors still induced data bias. Error checking was used to ensure data reliability by the BBS Taiwan workgroup.

This study provides environmental information about bird diversity. Three features of landscape habitats were identified to have an effect on bird diversity: (1) forestland decreased bird numbers, except for windbreak forests; (2) natural and farmland-related habitats had a higher effect on bird species richness; and (3) natural habitats had a higher effect on bird species evenness than farmland-related and urban habitats. Bird species richness and evenness were the main dissimilarities between natural and human-related habitats. Urban greenspaces could not replace the effect of forestland on bird species richness and evenness. Forestland can be used to predict bird species richness and evenness. Mixed trees, conifer forests, and bamboo forests were important forest characteristics for promoting species richness and evenness. In other landscape habitats, tall grasslandq and orchards were essential habitats for promoting both higher bird species richness and evenness.

## Method

### Data sources

The data from the 2010–2016 Taiwan Breeding Bird Survey (BBS Taiwan) were used to analyze the relationship between landscape habitat and bird ecology. The details of BBS Taiwan have been described in the past reference^35–38^, including sampling design, survey methods, and coverage of species in the dataset. BBS Taiwan is a nationwide monitoring project to survey breeding bird populations; the project started in 2009. The covered study areas were Taiwan island and Orchid Island, an area of 36,190 km^2^, composed of 70% mountains, with a humid subtropical and tropical monsoon climate.

**Figure 1:**
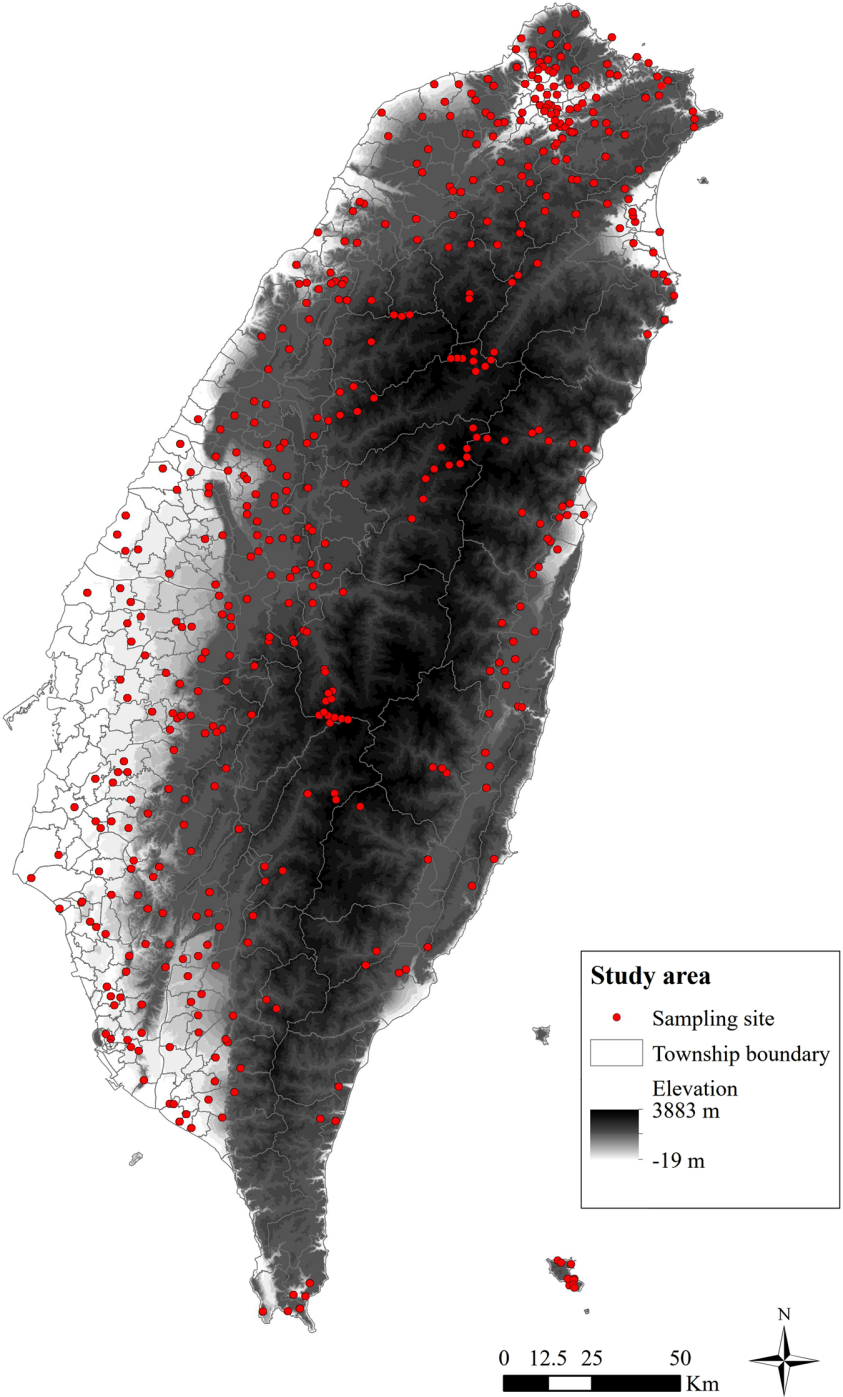
A total of 481 pre-selected and non-preselected sampling sites in the study area.

Two types of sampling plots were used: preselected sampling plots and non-preselected sampling plots. The study area was divided into 91 strata to cover various environmental gradients and habitats in the preselected sampling plots, considering 41 eco-regions and three elevations (0 to 1,000 m, 1,000 to 2,500 m, and 2,500 to 4,000 m). Stratified random sampling was used to determine 450 pre-selected sampling sites to cover 5% of the study area. Each sampling site is a 1 km × 1 km grid area. Vehicle accessibility was considered for long-term monitoring. For non-preselected sampling plots, volunteer surveyors established a sampling plot. A total of 481 pre-selected and non-preselected sampling sites are shown in Fig. 1.

The point-count method was used to survey bird species and numbers and to evaluate bird diversity, including resident birds and migrating birds. The point-count has three advantages: (1) not limited by Taiwan’s various mountain roads; (2) easy to clarify the relationship between birds and the environment; and (3) easy to control survey time for each sampling point^35^. Each sampling site consisted of six to ten sampling points within a 100 m radius. The interval straight-line distance was at least 200 m between sampling points to avoid duplicate records. All sampling sites were surveyed twice a year by volunteer surveyors during Taiwan’s main bird breeding season (March to June). The interval period was at least two weeks. Bird point counts lasted 6 minutes and were conducted within 4 hours after sunrise at each sampling point. The 6-minute point-count duration is sufficient to record over 80% of bird species in the breeding season^39^. For high-quality data, field excursions and training workshops were held to ensure that surveyors fully understood the survey method. The location of survey points, the correctness of the time period and distance, rare or easily misidentified species, and unusually high numbers were checked by the BBS Taiwan workgroup. Bird species and numbers were recorded for each survey sampling point. The taxonomic system of bird species is based on the Checklist of Birds of Taiwan by the Bird Record Committee of the Chinese Wild Bird Federation^40^.

### Independent variable: the type of landscape habitat

Landscape habitat type was divided into seven categories: forestland, farmland, grassland, freshwater wetland, aquaculture pond and saltpan, coastland, and building area. Landscape habitat types were divided into 26 sub-types. Forestland included 6 sub-types: broadleaf forest, conifer forest, mixed conifer–broadleaf forest (broadleaf forest >10% and conifer forest >10%), bamboo forest, mixed bamboo–broadleaf forest (broadleaf forest >10% and bamboo forest >10%), and windbreak forest (e.g., casuarina windbreak). Grassland included 5 sub-types: tall grassland (height >0.5 m), low grassland (height <0.5 m), high marsh (height >0.5 m), low marsh (height <0.5 m), and bamboo grassland. Freshwater wetlands included four sub-types: water storage area, lake, river (water surface width >3 m), and stream (water surface width <3 m). Coastland included two sub-types: intertidal (e.g., tidal mudflat and rock coast) and shoreline (e.g., rocky and sandy shoreline). Farmland included three sub-types: aquatic farmland (e.g., paddy, water chestnut, and lotus), dry farmland (e.g., vegetable, sugarcane, pineapple, and peanut), and orchard (e.g., fruit trees and nursery). Aquaculture pond and saltpan included three sub-types: flooded field, dried field, and abandoned field. The building area included three sub-types: urban park and greenspace, urban building area, and rural building area.

The surveyors recorded one or two main landscape habitat types and sub-types within a 100 m radius for each survey sampling point because most sampling points included more than one landscape habitat type and sub-type. Therefore, the number of landscape habitat types and sub-types were higher than the number of sampling points (30,656) (Table 2). The total percentage of landscape habitat types and sub-types were also higher than 100%. The two main landscape types were introduced into the dummy variable in the data analysis.

### Dependent variable: bird diversity

Species number and Margalef Richness Index are common species richness indexes^41^. The Pielou Evenness Index is a common species evenness index based on the evenness of the distribution of importance between species^41^. Although the Shannon-Wiener diversity index combines species richness and evenness and provides heterogeneity information for ecological studies^20,41^, the separate effects of species richness and evenness are difficult to distinguish. Therefore, four ecological indexes were used to address the purposes of this study: number of bird individuals (N), number of species (S), Margalef Richness Index (d), and Pielou Evenness Index (J’). Diversity indexes were calculated as

$${\rm{d}}=({\rm{S}}-1)/{\rm{lnN}};$$

$${\rm{J}}^{\prime} ={\rm{H}}^{\prime} /{\rm{lnS}};\,{\rm{H}}^{\prime} =-\,\mathop{\sum }\limits_{n=1}^{s}{P}_{i}\,\mathrm{ln}\,{P}_{i};$$

where S is the number of bird species; Pi is the density proportion of i bird species; N is the number of bird individuals^41–49^.

### Control variables

Wind and weather were recorded for each survey sampling point as bird detection was easily affected by climate factors. Wind force included four levels based on the Beaufort wind force scale: calm or light air (less than 1.6 m/s), light/gentle breeze (1.6–5.4 m/s), moderate/fresh breeze (5.5–10.7 m/s), and strong breeze (more than 10.8 m/s)^50^. Weather categories included sunny, cloudy, overcast, dense fog, and light rain. The weather category was recorded into the dummy variable in the data analysis.

### Data analysis

Hierarchical multiple regression analysis was used to analyze the association between the habitat types and sub-types and the four bird ecological indexes. First, the control variables were entered in model 1, including wind and weather. Second, the independent variables were entered in model 2 through dummy variables, including the habitat types and sub-types. The same process was performed for each hierarchical multiple regression analysis. The only difference was the dependent variable, including number of bird individuals (N), number of species (S), Margalef Richness Index (d), and Pielou Evenness Index (J’). Cook’s D statistic of less than 1.0 was used to inspect the influence of potential outliers in these models. No potential problems with multicollinearity were observed through assessments of the tolerance and variance inflation factor (VIF). Significant standardized beta coefficients and model changes were reported. All analyses were performed with SPSS Version 22.

## Supplementary information

Supplementary Table S1.

## Data Availability

The data that support the findings of this study are available from the Taiwan Breeding Bird Survey, but restrictions apply: the data were used under license for the current study and are not publicly available. Data are however available from the authors upon reasonable request and with permission of Taiwan Breeding Bird Survey.
