# Supplementary material for: Different Habitat Types Affect Bird Richness and Evenness
Source: Sci Rep. 2020 Jan 27;10:1221. doi: 10.1038/s41598-020-58202-4 (PMC6985263; doi:10.1038/s41598-020-58202-4)
Supplement: Supplementary file 1 — Supplementary Table S1. [file 41598_2020_58202_MOESM1_ESM.pdf]

Different Habitat Types Affect Bird Richness and Evenness

Hung-Ming Tu, Meng-Wen Fan, Jerome Chie-Jen Ko

[1]Department of Horticulture, National Chung Hsing University, Taichung, 40227, Taiwan

[2]Endemic Species Research Institute, Nantou, 55244, Taiwan

[*]hmtu@dragon.nchu.edu.com

Supplementary Table S1. List of the 295 bird species in this study. N: Non-breeding migratory birds; B: Breeding birds.

NO.	English name	Latin name	Family	Status in Taiwan	Frequency of occurrence (%)	Total counts
001	Light-vented Bulbul	*Pycnonotus sinensis*	Pycnonotidae	B	48.413	66,633
002	Eurasian Tree Sparrow	*Passer montanus*	Passeridae	B	28.681	66,011
003	Black Bulbul	*Hypsipetes leucocephalus*	Pycnonotidae	B	31.703	28,386
004	Red Collared-Dove	*Streptopelia tranquebarica*	Columbidae	B	18.707	27,088
005	Japanese White-eye	*Zosterops japonicus*	Zosteropidae	N/B	21.274	26,201
006	Barn Swallow	*Hirundo rustica*	Hirundinidae	N/B	10.238	14,735
007	Taiwan Barbet	*Psilopogon nuchalis*	Megalaimidae	B	24.623	14,136
008	Taiwan Scimitar-Babbler	*Pomatorhinus musicus*	Timaliidae	B	21.315	14,123
009	Morrison's Fulvetta	*Alcippe morrisonia*	Pellorneidae	B	14.121	13,406
010	Pacific Swallow	*Hirundo tahitica*	Hirundinidae	N/B	12.048	12,822
011	Javan Myna	*Acridotheres javanicus*	Sturnidae	N	12.321	11,299
012	Spotted Dove	*Streptopelia chinensis*	Columbidae	B	17.155	11,100
013	Gray Treepie	*Dendrocitta formosae*	Corvidae	B	17.426	11,040
014	Rufous-capped Babbler	*Cyanoderma ruficeps*	Timaliidae	B	20.495	10,538
015	Plain Prinia	*Prinia inornata*	Cisticolidae	B	15.651	9,149
016	Taiwan Yuhina	*Yuhina brunneiceps*	Zosteropidae	B	8.056	9,045
017	Styan's Bulbul	*Pycnonotus taivanus*	Pycnonotidae	B	6.536	8,468
018	Black-naped Monarch	*Hypothymis azurea*	Monarchidae	B	16.605	8,278
019	Cattle Egret	*Bubulcus ibis*	Ardeidae	N/B	3.155	7,439
020	House Swift	*Apus nipalensis*	Apodidae	B	3.572	7,382
021	Dusky Fulvetta	*Schoeniparus brunneus*	Pellorneidae	B	12.754	7,266
022	White-eared Sibia	*Heterophasia auricularis*	Leiothrichidae	B	8.097	7,238
023	Steere's Liocichla	*Liocichla steerii*	Leiothrichidae	B	8.047	6,919
024	Yellow-bellied Prinia	*Prinia flaviventris*	Cisticolidae	B	11.815	6,870
025	Little Egret	*Egretta garzetta*	Ardeidae	N/B	5.976	6,411
026	Black Drongo	*Dicrurus macrocercus*	Dicaeidae	N/B	10.581	5,888
027	Striated Swallow	*Cecropis striolata*	Hirundinidae	B	4.605	5,392
028	Rock Pigeon	*Columba livia*	Columbidae	N	1.962	5,305
029	Nutmeg Mannikin	*Lonchura punctulata*	Estrildidae	B	3.572	5,283
030	Taiwan Bamboo-Partridge	*Bambusicola sonorivox*	Phasianidae	B	9.341	5,241
031	Common Myna	*Acridotheres tristis*	Sturnidae	N	4.268	3,484
032	Zitting Cisticola	*Cisticola juncidis*	Cisticolidae	N/B	5.035	3,258
033	Rufous-faced Warbler	*Abroscopus albogularis*	Cettiidae	B	6.514	3,064
034	Black-crowned Night-Heron	*Nycticorax nycticorax*	Ardeidae	N/B	2.528	3,037
035	Asian House-Martin	*Delichon dasypus*	Hirundinidae	B	0.636	2,991
036	Black-necklaced Scimitar-Babbler	*Megapomatorhinus erythrocnemis*	Timaliidae	B	6.599	2,840
037	Eurasian Moorhen	*Gallinula chloropus*	Rallidae	B	3.855	2,825
038	White-bellied Erpornis	*Erpornis zantholeuca*	Vireonidae	B	4.936	2,780
039	Gray-chinned Minivet	*Pericrocotus solaris*	Campephagidae	B	4.446	2,524
040	Bronzed Drongo	*Dicrurus aeneus*	Dicaeidae	B	4.669	2,478
041	Yellowish-bellied Bush-Warbler	*Horornis acanthizoides*	Cettiidae	B	3.762	2,415
042	Black-throated Tit	*Aegithalos concinnus*	Aegithalidae	B	1.568	2,270
043	White-tailed Robin	*Cinclidium leucurum*	Muscicapidae	B	5.000	2,250
044	Collared Finchbill	*Spizixos semitorques*	Pycnonotidae	B	3.858	2,235
045	White-whiskered Laughingthrush	*Trochalopteron morrisonianum*	Leiothrichidae	B	2.862	2,015

NO.	English name	Latin name	Family	Status in Taiwan	Frequency of occurrence (%)	Total counts
046	Gray-throated Martin	*Riparia chinensis*	Hirundinidae	B	1.279	1,885
047	Western/Eastern Yellow Wagtail	*Motacilla flava/tschutschensis*	Motacillidae	N	1.724	1,778
048	White-rumped Munia	*Lonchura striata*	Estrildidae	B	1.447	1,751
049	Taiwan Blue-Magpie	*Urocissa caerulea*	Corvidae	B	1.902	1,616
050	Green-backed Tit	*Parus monticolus*	Paridae	B	3.018	1,577
051	Oriental Turtle-Dove	*Streptopelia orientalis*	Columbidae	N/B	2.614	1,443
052	Oriental Skylark	*Alauda gulgula*	Alaudidae	B	2.004	1,400
053	Flamecrest	*Regulus goodfellowi*	Regulidae	B	2.045	1,364
054	White-browed Bush-Robin	*Tarsiger indicus*	Muscicapidae	B	1.975	1,261
055	Black-winged Stilt	*Himantopus himantopus*	Recurvirostridae	N/B	0.550	1,250
056	Gray-capped Woodpecker	*Dendrocopos canicapillus*	Picidae	B	3.203	1,247
057	Little Ringed Plover	*Charadrius dubius*	Charadriidae	N/B	0.964	1,133
058	Brown Shrike	*Lanius cristatus*	Laniidae	N	3.174	1,117
059	White-browed Shortwing	*Brachypteryx montana*	Muscicapidae	B	2.134	1,114
060	Vivid Niltava	*Niltava vivida*	Muscicapidae	B	2.592	1,063
061	Collared Bush-Robin	*Tarsiger johnstoniae*	Muscicapidae	B	2.322	1,039
062	Wood Sandpiper	*Tringa glareola*	Scolopacidae	N	0.582	1,024
063	Taiwan Whistling-Thrush	*Myophonus insularis*	Muscicapidae	B	2.589	1,014
064	Eurasian Wren	*Troglodytes troglodytes*	Troglodytidae	B	1.517	951
065	White Wagtail	*Motacilla alba*	Motacillidae	N/B	2.223	945
066	Taiwan Fulvetta	*Fulvetta formosana*	Sylviidae	B	1.606	933
067	Eurasian Magpie	*Pica pica*	Corvidae	N	1.743	931
068	Taiwan Hwamei	*Garrulax taewanus*	Leiothrichidae	B	1.931	916
069	Vinous-throated Parrotbill	*Sinosuthora webbiana*	Sylviidae	B	0.786	876
070	Brownish-flanked Bush-Warbler	*Horornis fortipes*	Cettiidae	N/B	1.807	827
071	Taiwan Bush-Warbler	*Locustella alishanensis*	Locustellidae	B	1.698	753
072	Coal Tit	*Periparus ater*	Paridae	B	1.418	753
073	Little Grebe	*Tachybaptus ruficollis*	Podicipedidae	N/B	0.833	752
074	Large-billed Crow	*Corvus macrorhynchos*	Corvidae	B	1.482	732
075	Lesser Coucal	*Centropus bengalensis*	Cuculidae	B	1.902	716
076	Great Egret	*Ardea alba*	Ardeidae	N/B	0.662	681
077	Taiwan Cupwing	*Pnoepyga formosana*	Pnoepygidae	B	1.482	663
078	Maroon Oriole	*Oriolus traillii*	Oriolidae	B	1.511	649
079	White-breasted Waterhen	*Amaurornis phoenicurus*	Rallidae	B	1.441	608
080	Crested Serpent-Eagle	*Spilornis cheela*	Accipitridae	B	1.517	602
081	Oriental Cuckoo	*Cuculus optatus*	Cuculidae	B	1.673	597
082	Ring-necked Pheasant	*Phasianus colchicus*	Phasianidae	N/B	1.161	571
083	Taiwan Partridge	*Arborophila crudigularis*	Phasianidae	B	1.320	566
084	Taiwan Rosefinch	*Carpodacus formosanus*	Fringillidae	B	1.205	555
085	Gray Wagtail	*Motacilla cinerea*	Motacillidae	N	1.422	547
086	Crested Myna	*Acridotheres cristatellus*	Sturnidae	B	0.646	547
087	Green-winged Teal	*Anas crecca*	Anatidae	N	0.092	542
088	Gray Heron	*Ardea cinerea*	Ardeidae	N	0.487	537
089	Golden-headed Cisticola	*Cisticola exilis*	Cisticolidae	B	1.183	532
090	White-bellied Pigeon	*Treron sieboldii*	Columbidae	B	1.091	523
091	Black-collared Starling	*Gracupica nigricollis*	Sturnidae	N	0.821	511
092	Pale Thrush	*Turdus pallidus*	Turdidae	N	1.085	476
093	Plumbeous Redstart	*Phoenicurus fuliginosus*	Muscicapidae	B	1.062	475
094	Eastern Spot-billed Duck	*Anas zonorhyncha*	Anatidae	N/B	0.401	458
095	Common Sandpiper	*Actitis hypoleucos*	Scolopacidae	N	0.967	455
096	Black-faced Bunting	*Emberiza spodocephala*	Emberizidae	N	0.697	450
097	Long-tailed Shrike	*Lanius schach*	Laniidae	B	1.116	438
098	Common Kingfisher	*Alcedo atthis*	Alcedininae	N/B	1.180	429
099	Fire-breasted Flowerpecker	*Dicaeum ignipectus*	Dicaeidae	B	1.024	417
100	Asian Emerald Dove	*Chalcophaps indica*	Columbidae	B	1.116	408
101	Oriental Pratincole	*Glareola maldivarum*	Glareolidae	B	0.296	396
102	Rusty Laughingthrush	*Ianthocincla poecilorhyncha*	Leiothrichidae	B	0.623	360

NO.	English name	Latin name	Family	Status in Taiwan	Frequency of occurrence (%)	Total counts
103	Malayan Night-Heron	*Gorsachius melanolophus*	Ardeidae	B	0.960	356
104	Golden Parrotbill	*Suthora verreauxi*	Sylviidae	B	0.258	345
105	Pacific Golden-Plover	*Pluvialis fulva*	Charadriidae	N	0.130	327
106	Snowy-browed Flycatcher	*Ficedula hyperythra*	Muscicapidae	B	0.671	314
107	Intermediate Egret	*Mesophoyx/Ardea intermedia*	Ardeidae	N/B	0.557	312
108	Brown-headed Thrush	*Turdus chrysolaus*	Turdidae	N	0.566	308
109	Striated Prinia	*Prinia crinigera*	Cisticolidae	B	0.782	301
110	Pacific Swift	*Apus pacificus*	Apodidae	N	0.156	284
111	Crested Goshawk	*Accipiter trivirgatus*	Accipitridae	B	0.760	274
112	Domestic duck	*Anas platyrhynchos domesticus*	Anatidae	B	0.146	266
113	Eurasian Nuthatch	*Sitta europaea*	Sittidae	B	0.598	265
114	Gray-headed Bullfinch	*Pyrrhula erythaca*	Fringillidae	B	0.499	263
115	Yellow-browed Warbler	*Phylloscopus inornatus*	Phylloscopidae	N	0.636	251
116	Eurasian Jay	*Garrulus glandarius*	Corvidae	B	0.534	243
117	Chestnut Munia	*Lonchura atricapilla*	Estrildidae	N/B	0.169	239
118	Chinese x Taiwan Hwamei (hybrid)	*Garrulax canorus x taewanus*	Leiothrichidae	B	0.493	234
119	Taiwan Barwing	*Actinodura morrisoniana*	Leiothrichidae	B	0.398	228
120	Ashy Wood-Pigeon	*Columba pulchricollis*	Columbidae	B	0.452	224
121	Whiskered Tern	*Chlidonias hybrida*	Laridae	N	0.045	220
122	Brown-eared Bulbul	*Hypsipetes amaurotis*	Pycnonotidae	N/B	0.184	220
123	Japanese Bush-Warbler (borealis)	*Horornis diphone borealis*	Cettiidae	N	0.598	218
124	Red-necked Stint	*Calidris ruficollis*	Scolopacidae	N	0.013	212
125	Ferruginous Flycatcher	*Muscicapa ferruginea*	Muscicapidae	B	0.452	207
126	Kentish Plover	*Charadrius alexandrinus*	Charadriidae	N/B	0.137	196
127	Yellow Tit	*Machlolophus holsti*	Paridae	B	0.429	190
128	Sacred Ibis	*Threskiornis aethiopicus*	Threskiornithidae	N	0.105	183
129	Common Greenshank	*Tringa nebularia*	Scolopacidae	N	0.229	180
130	Red-billed Starling	*Spodiopsar sericeus*	Sturnidae	N	0.032	175
131	Brown Bullfinch	*Pyrrhula nipalensis*	Fringillidae	B	0.277	173
132	White-rumped Shama	*Copsychus malabaricus*	Muscicapidae	N	0.458	167
133	Manchurian Bush-Warbler	*Horornis borealis*	Scotocercidae	N	0.420	154
134	Arctic Warbler	*Phylloscopus borealis*	Phylloscopidae	N	0.420	151
135	Greater Painted-snipe	*Rostratula benghalensis*	Rostratulidae	B	0.229	145
136	Large Hawk-Cuckoo	*Hierococcyx sparverioides*	Cuculidae	B	0.398	144
137	Plain Flowerpecker	*Dicaeum minullum*	Dicaeidae	B	0.356	140
138	Eurasian Nutcracker	*Nucifraga caryocatactes*	Corvidae	B	0.312	137
139	Blue Rock-Thrush	*Monticola solitarius*	Muscicapidae	N/B	0.398	135
140	Pheasant-tailed Jacana	*Hydrophasianus chirurgus*	Jacanidae	N/B	0.080	133
141	Besra	*Accipiter virgatus*	Accipitridae	B	0.359	130
142	Daurian Redstart	*Phoenicurus auroreus*	Muscicapidae	N	0.372	128
143	Marsh Sandpiper	*Tringa stagnatilis*	Scolopacidae	N	0.070	127
144	Common Snipe	*Gallinago gallinago*	Scolopacidae	N	0.089	122
145	Chestnut-bellied Tit	*Sittiparus castaneoventris*	Paridae	B	0.175	111
146	White-shouldered Starling	*Sturnia sinensis*	Sturnidae	N	0.089	106
147	Sharp-tailed Sandpiper	*Calidris acuminata*	Scolopacidae	N	0.038	96
148	Chestnut-tailed Starling	*Sturnia malabarica*	Sturnidae	N	0.137	96
149	Barred Buttonquail	*Turnix suscitator*	Turnicidae	B	0.213	95
150	Siberian Rubythroat	*Calliope calliope*	Muscicapidae	N	0.264	94
151	Rufous-crowned Laughingthrush	*Ianthocincla ruficeps*	Leiothrichidae	B	0.080	93
152	Island Thrush	*Turdus poliocephalus*	Turdidae	B	0.137	93
153	Eurasian Coot	*Fulica atra*	Rallidae	N	0.060	82
154	Fairy Pitta	*Pitta nympha*	Pittidae	B	0.219	81
155	Oriental Magpie-Robin	*Copsychus saularis*	Muscicapidae	N	0.223	80
156	Eurasian Siskin	*Spinus spinus*	Fringillidae	N	0.003	80
157	Asian Glossy Starling	*Aplonis panayensis*	Sturnidae	N	0.073	76
158	Russet Sparrow	*Passer rutilans*	Passeridae	B	0.105	74
159	Long-toed Stint	*Calidris subminuta*	Scolopacidae	N	0.022	72

NO.	English name	Latin name	Family	Status in Taiwan	Frequency of occurrence (%)	Total counts
160	Black-naped Oriole	*Oriolus chinensis*	Oriolidae	N/B	0.172	70
161	Gray-tailed Tattler	*Tringa brevipes*	Scolopacidae	N	0.048	69
162	Black-shouldered Kite	*Elanus caeruleus*	Accipitridae	B	0.181	66
163	Little Tern	*Sternula albifrons*	Laridae	B	0.067	65
164	Gray-faced Buzzard	*Butastur indicus*	Accipitridae	N	0.041	65
165	White-backed Woodpecker	*Dendrocopos leucotos*	Picidae	B	0.172	60
166	Cinnamon Bittern	*Ixobrychus cinnamomeus*	Ardeidae	B	0.137	56
167	Alpine Accentor	*Prunella collaris*	Prunellidae	B	0.118	56
168	Black Kite	*Milvus migrans*	Accipitridae	B	0.130	53
169	Common Redshank	*Tringa totanus*	Scolopacidae	N	0.089	52
170	Arctic Warbler (complex)	*Phylloscopus borealis (complex)*	Phylloscopidae	N	0.140	49
171	Black-throated Laughingthrush	*Ianthocincla chinensis*	Leiothrichidae	N	0.111	47
172	Yellow Bittern	*Ixobrychus sinensis*	Ardeidae	B	0.089	44
173	Swinhoe's Pheasant	*Lophura swinhoii*	Phasianidae	B	0.111	41
174	Muscovy duck	*Cairina moschata domestica*	Anatidae	B	0.089	41
175	Collared Owlet	*Glaucidium brodiei*	Strigidae	B	0.114	40
176	Northern Pintail	*Anas acuta*	Anatidae	N	0.013	39
177	Ruddy-breasted Crake	*Zapornia fusca*	Rallidae	B	0.095	37
178	Olive-backed Pipit	*Anthus hodgsoni*	Motacillidae	N	0.045	37
179	Green Sandpiper	*Tringa ochropus*	Scolopacidae	N	0.089	35
180	Oriental Honey-buzzard	*Pernis ptilorhynchus*	Accipitridae	N/B	0.083	33
181	Little Forktail	*Enicurus scouleri*	Muscicapidae	B	0.060	32
182	Eurasian Wigeon	*Anas penelope*	Anatidae	N	0.006	32
183	Ashy Minivet	*Pericrocotus divaricatus*	Campephagidae	N	0.041	32
184	Gray-faced Woodpecker	*Picus canus*	Picidae	B	0.099	31
185	Philippine Cuckoo-Dove	*Macropygia tenuirostris*	Columbidae	B	0.051	30
186	Orange-cheeked Waxbill	*Estrilda melpoda*	Estrildidae	N	0.019	30
187	Lowland White-eye	*Zosterops meyeni*	Zosteropidae	B	0.057	30
188	Lesser Black-backed Gull	*Larus fuscus*	Laridae	N	0.010	27
189	Great Cormorant	*Phalacrocorax carbo*	Phalacrocoracidae	N	0.032	27
190	Whistling Green-Pigeon	*Treron formosae*	Columbidae	B	0.048	26
191	Mallard	*Anas platyrhynchos*	Anatidae	N	0.019	25
192	Brown Dipper	*Cinclus pallasii*	Cinclidae	B	0.064	25
193	White's Thrush	*Zoothera aurea*	Turdidae	N	0.067	24
194	Lesser Sand-Plover	*Charadrius mongolus*	Charadriidae	N	0.025	24
195	Tufted Duck	*Aythya fuligula*	Anatidae	N	0.013	23
196	Savanna Nightjar	*Caprimulgus affinis*	Caprimulgidae	B	0.060	23
197	Oriental Reed-Warbler	*Acrocephalus orientalis*	Muscicapidae	N	0.054	23
198	Indian Silverbill	*Euodice malabarica*	Estrildidae	N	0.019	23
199	Red-throated Pipit	*Anthus cervinus*	Motacillidae	N	0.029	21
200	Northern Shoveler	*Anas clypeata*	Anatidae	N	0.013	21
201	Yellow-billed Grosbeak	*Eophona migratoria*	Fringillidae	N	0.003	20
202	Mikado Pheasant	*Syrmaticus mikado*	Phasianidae	B	0.041	20
203	Dusky Thrush	*Turdus eunomus*	Turdidae	N	0.041	19
204	White-winged Tern	*Chlidonias leucopterus*	Laridae	N	0.003	16
205	Ruddy Turnstone	*Arenaria interpres*	Scolopacidae	N	0.003	16
206	Japanese Paradise-Flycatcher	*Terpsiphone atrocaudata*	Monarchidae	N/B	0.041	16
207	Common Tern	*Sterna hirundo*	Laridae	N	0.006	16
208	Collared Scops-Owl	*Otus lettia*	Strigidae	B	0.035	16
209	Spotted Redshank	*Tringa erythropus*	Scolopacidae	N	0.006	15
210	Richard's Pipit	*Anthus richardi*	Motacillidae	N	0.032	14
211	Red Junglefowl	*Gallus gallus*	Phasianidae	B	0.022	14
212	Oriental Greenfinch	*Chloris sinica*	Fringillidae	N	0.003	14
213	Garganey	*Anas querquedula*	Anatidae	N	0.013	14
214	Osprey	*Pandion haliaetus*	Pandionidae	N	0.032	12
215	Jungle Myna	*Acridotheres fuscus*	Sturnidae	N	0.013	12
216	Gray-streaked Flycatcher	*Muscicapa griseisticta*	Muscicapidae	N	0.029	12

NO.	English name	Latin name	Family	Status in Taiwan	Frequency of occurrence (%)	Total counts
217	Brambling	*Fringilla montifringilla*	Fringillidae	N	0.006	12
218	Terek Sandpiper	*Xenus cinereus*	Scolopacidae	N	0.006	11
219	Slaty-breasted Rail	*Gallirallus striatus*	Rallidae	B	0.035	11
220	Dunlin	*Calidris alpina*	Scolopacidae	N	0.010	11
221	Chinese Pond-Heron	*Ardeola bacchus*	Ardeidae	N	0.032	11
222	Asian Stubtail	*Urosphena squameiceps*	Cettiidae	N	0.035	11
223	Swinhoe's Snipe	*Gallinago megala*	Scolopacidae	N	0.003	10
224	Striated Heron	*Butorides striata*	Ardeidae	N/B	0.019	10
225	Slaty-legged Crake	*Rallina eurizonoides*	Rallidae	B	0.022	10
226	Eurasian Kestrel	*Falco tinnunculus*	Falconidae	N	0.025	10
227	Common Waxbill	*Estrilda astrild*	Estrildidae	N	0.003	10
228	Chinese Egret	*Egretta eulophotes*	Ardeidae	N	0.019	10
229	Pacific Reef-Heron	*Egretta sacra*	Ardeidae	B	0.025	9
230	Large Cuckooshrike	*Coracina macei*	Campephagidae	B	0.010	9
231	Great myna	*Acridotheres grandis*	Sturnidae	N	0.016	9
232	White-cheeked Starling	*Spodiopsar cineraceus*	Sturnidae	N/B	0.006	8
233	Vinous-breasted starling	*Acridotheres burmannicus*	Sturnidae	N	0.010	8
234	Scaly Thrush	*Zoothera dauma*	Turdidae	B	0.016	8
235	Java Sparrow	*Lonchura oryzivora*	Estrildidae	N	0.006	8
236	Chinese Sparrowhawk	*Accipiter soloensis*	Accipitridae	N	0.013	8
237	Black Eagle	*Ictinaetus malaiensis*	Accipitridae	B	0.022	8
238	White-browed Laughingthrush	*Ianthocincla sannio*	Leiothrichidae	N	0.019	7
239	Japanese Bush-Warbler	*Horornis diphone*	Cettiidae	N	0.016	7
240	Chinese Hwamei	*Garrulax canorus*	Leiothrichidae	N	0.010	7
241	Black-faced Spoonbill	*Platalea minor*	Threskiornithidae	N	0.006	7
242	Bank Swallow	*Riparia riparia*	Hirundinidae	N	0.010	7
243	Silver-backed Needletail	*Hirundapus cochinchinensis*	Apodidae	B	0.003	6
244	Peregrine Falcon	*Falco peregrinus*	Falconidae	N/B	0.019	6
245	Pechora Pipit	*Anthus gustavi*	Motacillidae	N	0.003	6
246	Mountain Scops-Owl	*Otus spilocephalus*	Strigidae	B	0.016	6
247	Light-vented x Styan's Bulbul	*Pycnonotus taivanus x sinensis*	Pycnonotidae	B	0.016	6
248	Eyebrowed Thrush	*Turdus obscurus*	Turdidae	N	0.013	5
249	Domestic goose	*Anser anser domesticus*	Anatidae	B	0.010	5
250	Common Ringed Plover	*Charadrius hiaticula*	Charadriidae	N	0.006	5
251	Rose-ringed Parakeet	*Psittacula krameri*	Psittaculidae	N	0.006	4
252	Lesser Cuckoo	*Cuculus poliocephalus*	Cuculidae	N	0.013	4
253	Black-tailed Gull	*Larus crassirostris*	Laridae	N	0.003	4
254	Temminck's Stint	*Calidris temminckii*	Scolopacidae	N	0.006	3
255	Sulphur-crested Cockatoo	*Cacatua galerita*	Cacatuidae	N	0.010	3
256	Herring Gull	*Larus argentatus*	Laridae	N	0.003	3
257	Chestnut Bunting	*Emberiza rutila*	Emberizidae	N	0.003	3
258	Black-necked swan	*Cygnus melancoryphus*	Anatidae	N	0.006	3
259	Asian Brown Flycatcher	*Muscicapa dauurica*	Muscicapidae	N	0.010	3
260	Whimbrel	*Numenius phaeopus*	Scolopacidae	N	0.003	2
261	Trumpeter Hornbill	*Bycanistes bucinator*	Bucerotidae	N	0.006	2
262	Siberian Stonechat	*Saxicola maurus*	Muscicapidae	N	0.003	2
263	Pallas's Leaf Warbler	*Phylloscopus proregulus*	Phylloscopidae	N	0.006	2
264	Mandarin Duck	*Aix galericulata*	Anatidae	N/B	0.003	2
265	Gray-headed Lapwing	*Vanellus cinereus*	Charadriidae	N	0.003	2
266	European Starling	*Sturnus vulgaris*	Sturnidae	N	0.006	2
267	Eastern Crowned Leaf Warbler	*Phylloscopus coronatus*	Phylloscopidae	N	0.006	2
268	Eastern Buzzard	*Buteo japonicus*	Accipitridae	N	0.006	2
269	Dusky Warbler	*Phylloscopus fuscatus*	Phylloscopidae	N	0.006	2
270	Dollarbird	*Eurystomus orientalis*	Coraciidae	N	0.006	2
271	Citrine Wagtail	*Motacilla citreola*	Motacillidae	N	0.006	2
272	Caspian Tern	*Hydroprogne caspia*	Laridae	N	0.006	2
273	American Pipit	*Anthus rubescens*	Motacillidae	N	0.006	2

NO.	English name	Latin name	Family	Status in Taiwan	Frequency of occurrence (%)	Total counts
274	Yellow Bunting	*Emberiza sulphurata*	Emberizidae	N	0.003	1
275	Tristram's Bunting	*Emberiza tristrami*	Emberizidae	N	0.003	1
276	Ryukyu Scops-Owl	*Otus elegans*	Strigidae	B	0.003	1
277	Rustic Bunting	*Emberiza rustica*	Emberizidae	N	0.003	1
278	Ruff	*Calidris pugnax*	Scolopacidae	N	0.003	1
279	Red-flanked Bluetail	*Tarsiger cyanurus*	Muscicapidae	N	0.003	1
280	Red-breasted Flycatcher	*Ficedula parva*	Muscicapidae	N	0.003	1
281	Red-billed Blue-Magpie	*Urocissa erythroryncha*	Corvidae	N	0.003	1
282	Purple Heron	*Ardea purpurea*	Ardeidae	N/B	0.003	1
283	Pied Avocet	*Recurvirostra avosetta*	Recurvirostridae	N	0.003	1
284	Northern Goshawk	*Accipiter gentilis*	Accipitridae	N	0.003	1
285	Northern Boobook	*Ninox japonica*	Strigidae	N/B	0.003	1
286	Indian Cuckoo	*Cuculus micropterus*	Cuculidae	N	0.003	1
287	Himalayan Owl	*Strix nivicolum*	Strigidae	B	0.003	1
288	Glossy Ibis	*Plegadis falcinellus*	Threskiornithidae	N	0.003	1
289	Eurasian Skylark	*Alauda arvensis*	Alaudidae	N	0.003	1
290	Curlew Sandpiper	*Calidris ferruginea*	Scolopacidae	N	0.003	1
291	Common Hill Myna	*Gracula religiosa*	Sturnidae	N	0.003	1
292	Black-winged Cuckooshrike	*Lalage melaschistos*	Campephagidae	N	0.003	1
293	Black-tailed Godwit	*Limosa limosa*	Scolopacidae	N	0.003	1
294	Azure-winged Magpie	*Cyanopica cyanus*	Corvidae	N	0.003	1
295	Asian Koel	*Eudynamys scolopaceus*	Cuculidae	N	0.003	1
					Total	540,254
